# Comparative analysis of intestinal and reproductive function in older laying hens with three egg laying levels

**DOI:** 10.3389/fmicb.2025.1582516

**Published:** 2025-07-03

**Authors:** Taoyan Qiang, Keying Zhang, Qiufeng Zeng, Xuemei Ding, Shiping Bai, Yan Liu, Shengyu Xu, Yue Xuan, Shanshan Li, Jianping Wang

**Affiliations:** Key Laboratory of Animal Disease-Resistance Nutrition, Ministry of Agriculture and Rural Affairs, Key Laboratory of Sichuan Province, Animal Nutrition Institute, Sichuan Agricultural University, Chengdu, China

**Keywords:** different egg-laying rates, production performance, egg quality, intestinal function, ovarian function

## Abstract

**Introduction:**

In production, older laying hens exhibit different levels of egg production, though these hens with the same genetic background, age, and feeding conditions. It is speculated that this may be due to varying intestinal function in laying hens with different egg production levels. To verify this speculation, this experiment aims to compare the intestinal function in laying hens with different egg production levels.

**Methods:**

After a 6-week pre-feeding with 1,000 hens, aged 60 weeks, we observed that the overall egg-laying rate for the flock was roughly 85%. Based on the data, a total of 120 healthy Lohmann Pink laying hens, aged 66 weeks, were selected for the experiment, which established three treatment groups, low egg-laying rate (LR, 76.89% ± 1.65%), medium egg-laying rate (MR, 84.96% ± 1.01%), and high egg-laying rate (HR, 93.12% ± 1.73%).

**Results:**

The results indicated that the HR group exhibited significantly enhanced egg production, egg mass, feed efficiency, and qualified egg rate over the 12-week period (*p* < 0.05). Egg quality parameters, including Haugh units and eggshell strength, were also markedly elevated in the HR group (*p* < 0.05). Additionally, ovarian tissues of HR group demonstrated substantially increased activities of antioxidant enzymes, such as catalase (CAT), total antioxidant capacity (T-AOC), and glutathione (GSH) (*p* < 0.05). Concurrently, the mRNA expression of the antioxidant gene nuclear factor erythroid 2-related factor 2 (*Nrf2*) in both ovarian and reproductive tract tissues was significantly upregulated, while the expression of the pro-apoptotic gene cysteinyl aspartate 3 (*Caspase 3*) was downregulated (*p* < 0.05). Regarding intestinal health, the jejunal mucosa in the HR group displayed elevated mRNA expression of intestinal barrier-related genes, including claudin 1 (*CLDN1*) and mucin-2 (*MUC-2*) (*p* < 0.05). Cecal microbiota analysis further indicated that the HR group exhibited a higher relative abundance of *Firmicutes* (phylum), *Faecalibacterium* (genus), and *Lactobacillus* (genus) (*p* < 0.05), whereas the abundance of *Proteobacteria* (phylum) and *Actinobacteriota* (phylum) was significantly reduced (*p* < 0.01). Notably, alpha diversity indices of the cecal microbiota (observed OTUs, Shannon, Simpson, and Chao1 indices) were markedly lower in the HR group (*p* < 0.05). Correlation analysis highlighted a positive association between the abundance of *Bacillus* (genus) and egg mass.

**Discussion:**

These results suggest that hens with higher egg-laying rates demonstrated enhanced production performance and egg quality, which may be attributed to better intestine health (better barrier function, higher enrichment of *Faecalibacterium* and *Lactobacillus* in the cecum) and reproductive system health (improved antioxidant capacity). Additionally, the *SIRT1*-related apoptosis pathway and *Nrf2* signaling pathway were associated with the enhancement of ovarian function in this study.

## Graphical abstract

The differences of high laying hens with low laying hens under the same conditions (genetic background, age, diet, environment). The up arrow (↑,+) and down arrow (↓,−) represent up-regulation and down-regulation of enzyme activities and gene expression in layers at higher egg-laying level.
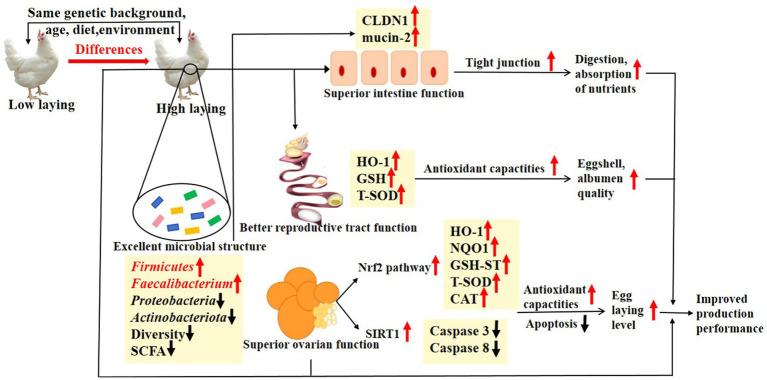

## Introduction

Aging in laying hens is a multifaceted physiological process characterized by progressive declines in reproductive efficiency (Liao et al., 2023). By 72–80 weeks of age, commercial laying hens often exhibit marked variability in egg production rates, with up to 30% of flocks experiencing premature culling due to inconsistent lay cycles. In terms of layer production, this aging process can contribute to a deterioration in the physiological capabilities of laying hens (Qiang et al., 2023; Xu et al., 2023). This reduction in physiological capabilities leads to decreased production performance, which serves a significant reason for the premature culling of laying hens in their later phases (He et al., 2023). Production observations have shown that laying hens, despite sharing the same genetic background, age, and feeding conditions, displayed differing levels of egg production during the later laying stage. We speculated that these variations might be linked to different extents of physiological aging among hens at various egg-laying levels.

Aged hens face a constellation of physiological challenges, including intestinal dysfunction, oxidative stress accumulation, and reproductive tract degeneration, all of which converge to impair egg synthesis and quality. The intestine serves as the primary organ for digestion and absorption in animals, significantly contributing to the processes of digestion and transformation in hens (Gu et al., 2021). A decline in intestinal function occurs naturally in older hens (Drozdowski and Thomson, 2006). Consequently, even under identical conditions such as genetic background, age, nutrition, management, and environment, laying hens demonstrate varying levels of egg production. We hypothesized that this variation may stem from differing extents of intestinal aging in the hens. The impact of aging on intestinal function primarily involves alterations in intestinal morphology, a reduction in antioxidant capacity, and changes in the composition of microbial species present in laying hens (Biagi et al., 2013; Celi et al., 2017; Li et al., 2022). Alterations in intestinal morphology are chiefly reflected in a weakened intestinal barrier function (Soenen et al., 2016). A large amount of oxidative stress can occur during the aging process of animals, which may cause more oxidative damage in hens (Liu et al., 2018; Gu et al., 2021). Additionally, oxidative stress can cause intestinal inflammatory diseases (Li and Chang, 2021), which may keep the intestines in a prolonged unhealthy state and affect the nutrient absorption from the feed (Gessner et al., 2017). Similarly, oxidative damage in the magnum and uterus organs responsible for albumen secretion and eggshell calcification could directly reduce egg weight and eggshell strength (Jing et al., 2014). The intestinal microbiota was important to the immune system in poultry (Bauer et al., 2006). With advancing age, the quantity, composition, and activity of the intestinal microbiota in laying hens can change (Cao et al., 2020). Hence, it may also influence their production performance of hens. Studies indicated that including antioxidants, such as rutin and resveratrol, which can improve intestinal health of older hens (Ding et al., 2022; Li et al., 2022). However, the connection between different egg-laying levels in older hens (under the same conditions) and the related decline in intestinal function (including digestion, absorption, and transformation) has not yet been explored. The decline in egg production and quality in aging layers stems from complex interactions among oxidative stress, apoptosis, ovarian function, gut health, and oviduct (magnum and uterus) performance. Oxidative stress acts as a central mediator, where excessive ROS accumulation damages ovarian granulosa cells, impairing steroidogenesis and follicular development, while simultaneously triggering apoptosis in both ovarian and oviduct tissues. Gut dysfunction exacerbates this cycle through impaired nutrient absorption and increased intestinal permeability, allowing endotoxin translocation that amplifies systemic inflammation and oxidative stress.

This research compared the variations in intestinal function among laying hens with different egg-laying levels. This comparison sought to alleviate the declined intestinal function in hens and enhance production performance. Ultimately, the research intended to offer a theoretical framework for harnessing the genetic potential of older laying hens.

## Materials and methods

### Animals and experimental design

Approval for all procedures was granted by the Animal Care and Use Committee at Sichuan Agricultural University. This study was funded by the National Key Research and Development Program of China. After a 6-week pre-feeding (same diet) with 1,000 hens, aged 60 weeks, we observed that the overall egg-laying rate for the flock was roughly 85%. Approximately 30% of the flock consisted of hens that exhibited an egg-laying rate of around 76%, those with a rate of approximately 85% accounted for about 50%, and those with a rate of about 93% accounted for around 20%. Based on the data, 120 layers (66 weeks old, average weight is 1.9 kilogram) were selected and divided into three groups (LR: 76.89 ± 1.65% vs. MR: 84.96% ± 1.01% vs. HR: 93.12% ± 1.73%). All experimental hens are Roman Pink and are housed in the middle and lower levels of a three-tier cage. Daily recordings of temperature and humidity are conducted to ensure consistency in the rearing environment. All experimental hens are provided with the same corn-soybean diet (Supplementary Table S1). Each group consists of 40 hens (10 replicates of 4 hens each). The duration of the experiment was 12 weeks. The temperature of the layer house at 22°C and a daily light cycle of 16 h and 8 h of darkness. Layers had ad libitum access to water, while feed intake was restricted to 120 grams per layer daily.

### Productive performance

The daily records included the counts of both qualified and unqualified eggs (with weights less than 50 g or greater than 75 g) for each replicate. The egg-laying rate was determined by summing the total number of eggs produced each day. Average daily production was calculated to express egg production. The feed conversion ratio (FCR) was derived from the total feed consumed (measured in grams) relative to the total weight of the eggs produced (also in grams). The rate of unqualified eggs was defined as the proportion of unqualified eggs to the total number of eggs laid for each replicate. Total egg mass was calculated by multiplying the egg-laying rate with the average weight of the eggs.

### Sample collection

Following a 12-week experimental period, a total of 30 laying hens (3 treatment groups with 10 replicates each, 1 hen per replicate) were individually weighed. Blood samples were taken from the cervical veins and transferred into sterile syringes. Centrifugation of the samples was carried out at a force of 3,000 × g for 15 min to isolate plasma and preserved at −20°C until analysis. When the layers were euthanized using CO_2_, samples of the ovary, uterus, and magnum of the oviduct were collected. Then, a portion of the intestinal segment was opened, the mucosa was scraped onto glass microscope slides and transferred into two eppendorf tubes. And the cecal chyme was collected and subsequently divided into three eppendorf tubes, where it was stored at −80°C for the purpose of analyzing the microbial 16S rRNA within the cecal chyme.

### Egg quality

At the conclusion of the study, 20 eggs were gathered per group (60 eggs) for the evaluation of egg parameters, the eggs collected immediately from hen house and assessed for egg quality. The strength of the eggshell was evaluated with an Egg Shell Force Gauge (model II, Robotmation Co., Ltd., Tokyo, Japan). To determine the average thickness of the eggshell, measurements were taken at three different points on each egg using an Egg Shell Thickness Gauge (Robotmation Co., Ltd.). The albumen quality was assessed utilizing an Egg Analyzer (EMT-7300, Robotmation Co., Ltd.). A colorimeter (3NH-NR20XE, China) was used to measure the lightness (L) of the eggshells. The calculation for the albumen ratio was performed as 100 × (albumen weight [g]/egg weight [g]).

### Antioxidant enzyme activity and related gene expression in ovary and reproductive tract

Following the collection of blood, the laying hens were euthanized using CO_2_, samples of the ovary, uterus, and magnum of the oviduct were collected, rinsed with saline, and transferred into eppendorf tubes. Some of these samples were preserved at −80°C for analyzing related genes in the ovary, uterus, and magnum of the oviduct, mainly including sirtuin 1 (*SIRT1*), nuclear factor erythroid2-related factor 2 (*Nrf2*), NAD(P)H: quinone oxidoreductase 1 (*NQO1*), heme oxygenase-1 (*HO-1*), B lymphoma 2 associated X protein (*Bax*), B-cell lymphoma-2 (*Bcl2*), cysteinyl aspartate 3 (*Caspase 3*), cysteinyl aspartate 8 (*Caspase 8*), cysteinyl aspartate 9 (*Caspase 9*). The genes and primer design are shown in Supplementary Table S2. The other samples were measured the antioxidant capacity, including catalase (CAT, A064-1-1), total antioxidant capacity (T-AOC, A015-2-1), glutathione (GSH, A006-2-1), glutathione S-transferas (GSH-ST, A004-1-1), glutathione peroxidase (GSH-Px, A005-1-1), total superoxide dismutase (T-SOD, A001-1), malondialdehyde (MDA, A003-2) (Nanjing Jiancheng Biotechnology Institute, Nanjing, China).

### DNA extraction and microbiota analysis

Ten replicate samples were randomly chosen to assess the expression levels of jejunal mucosal barrier-associated genes, including zonula occludens protein-1 (*ZO-1*), claudin1 (*CLDN1*), occludin (*OCLN*), and mucin-2 (*MUC-2*). The expression analysis was carried out using real-time quantitative PCR in a 384-well plate format. All kits were sourced from Takara Bio, Ltd. (Dalian, China), following the manufacturer’s guidelines. Additionally, β-actin served as the internal control. Details regarding the genes and primer design can be found in Supplementary Table S2.

For analysis of the microbial community, six replicate cecal chyme samples were randomly chosen from each treatment group and forwarded to to NovoMagic Biotechnology Co., Ltd. (Beijing, China) for 16S rRNA analysis of the microbial community. Mainly includes DNA extraction using kits, PCR amplification and library construction, high-throughput sequencing, raw data quality control, sequence assembly and denoising, species annotation, diversity analysis, and differential species analysis. OTU clustering and species annotation were performed based on the OTUs obtained from the clustering analysis, combined with the actual conditions of this experiment. The common and unique OTUs among the three treatments were analyzed. This was mainly achieved using the mapping function of the NovoMagic Cloud platform to create petal diagrams for visualization. The species annotation results facilitated the selection of the top 10 species with the highest abundance for both phylum and genus levels within each treatment group. Cumulative bar charts of relative species abundance were generated. Alpha diversity indices (Chao1, Simpson, etc.) were used to assess the complexity of the samples and were calculated using Qiime software (Version 1.7.0). Comparative analysis of multiple samples included Principal Co-ordinates Analysis (PCoA) and LDA effect size analysis (LEfSe) for comparing different treatment samples. The NovoMagic Cloud platform was used for Spearman analysis of environmental factors, primarily to analyze the correlation between the relative abundance of cecal chyme microbial genera and the egg-laying rate, and egg mass from weeks 1 to 12. The correlation *p*-values underwent false discovery rate (FDR) correction using the Benjamini-Hochberg procedure.

### Statistical analysis

Data were analyzed utilizing one-way ANOVA through the GLM procedure of SAS 9.4 software (SAS Institute, Cary, NC) alongside GraphPad Prism (GraphPad Inc., La Jolla, CA). The Tukey method was used for multiple comparisons, with a significance level set at *p* ≤ 0.05. Regarding the microbiota data, alpha and beta diversity were assessed with the Wilcox rank sum test and the weighted UniFrac distance matrices computed using QIIME (Version 1.7.0). Sample comparisons were conducted with PCoA and LEfSe.

## Results

### Production performance

**Figure 1:**
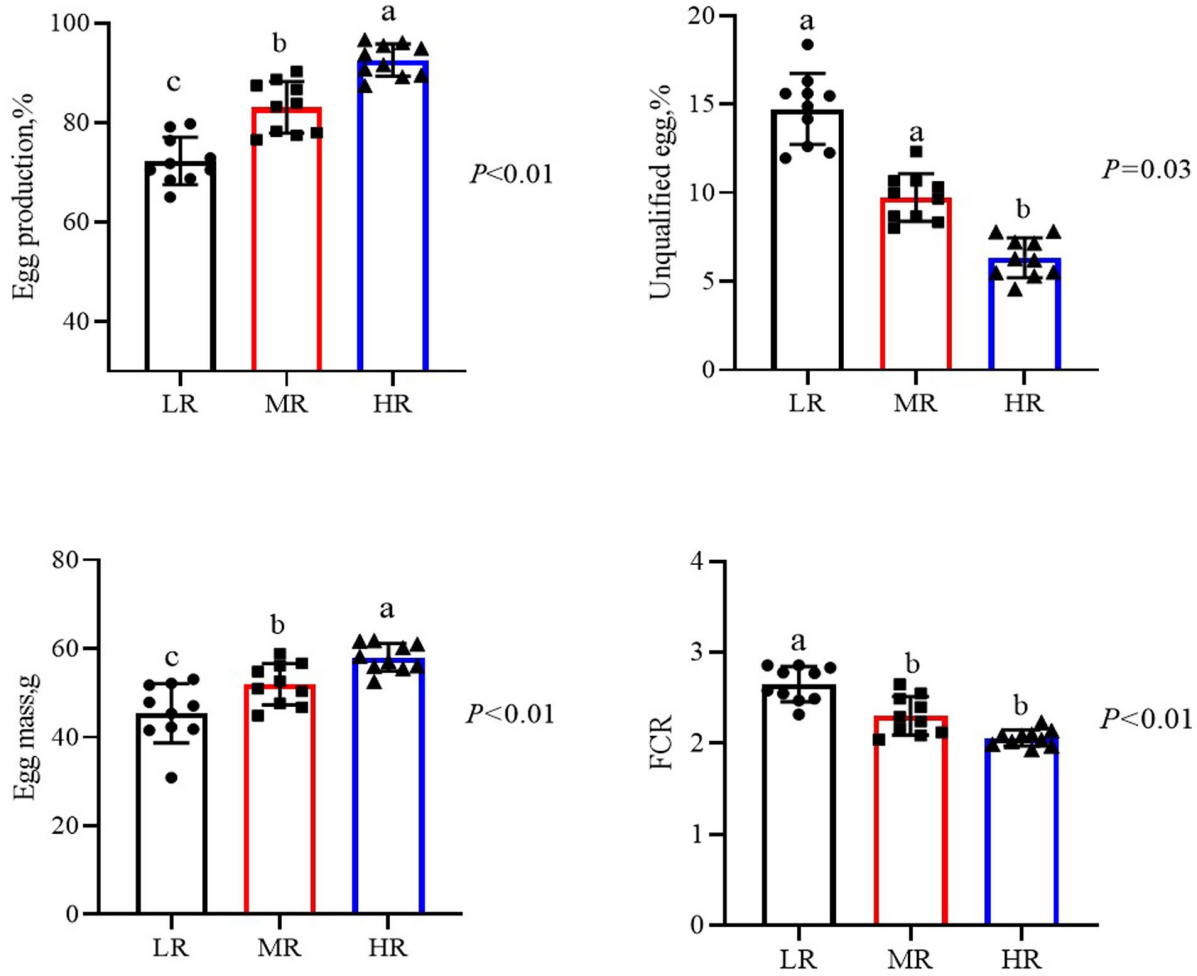
Differential analysis of production performance in laying hens with different egg laying rates during 1–12 weeks. LR, low egg laying rate; MR, normal egg laying rate; HR, high egg laying rate; FCR, feed conversion ratio. a, b means with different superscripts within a column differ significantly (*P* ≤ 0.05) (*n* = 10).

During the 12-week trial period, significant differences emerged in production parameters between three groups (Figure 1), the HR and MR groups exhibited significantly higher egg production, egg mass, and feed conversion efficiency compared to the LR group, with the HR having the highest values during the 12 weeks (*p* < 0.01). However, feed intake did not differ among three groups. Additionally, the unqualified egg rate was lower in the HR and MR groups than in the LR group (*p* < 0.05).

### Egg quality

**Figure 2:**
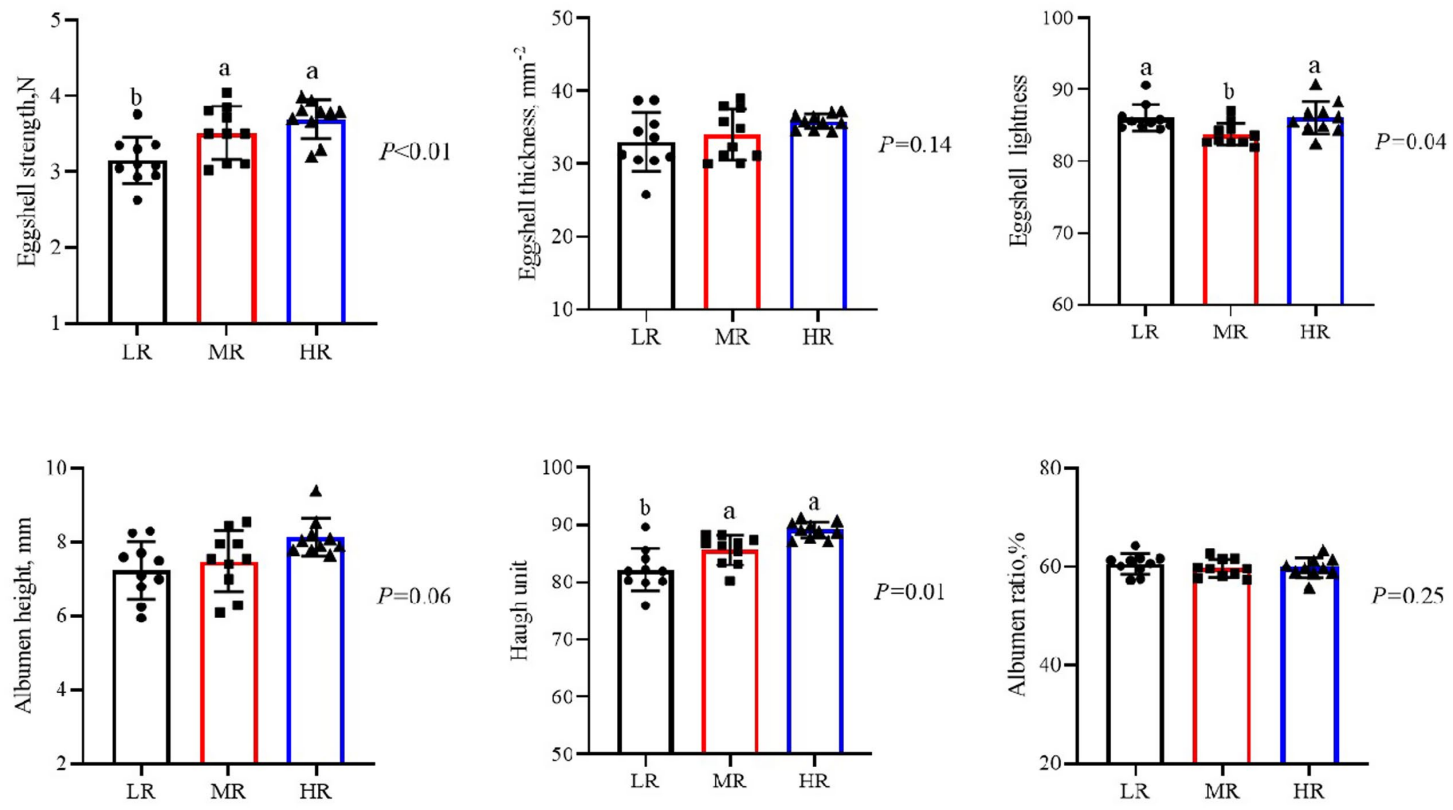
Differential analysis of 12 week egg quality in laying hens with different egg-laying rates. LR, low egg laying rate; MR, normal egg laying rate; HR, high egg laying rate. a, b means with different superscripts within a column differ significantly (*P* ≤ 0.05) (*n* = 10).

Egg quality analysis (Figure 2) showed that HR and MR groups presented higher eggshell strength (*p* < 0.05) and Haugh units (*p* < 0.05) compared to LR group. Additionally, the MR group demonstrated a lower eggshell lightness (a*) value at the 12th week (*p* < 0.05). In contrast, albumen height and eggshell thickness were not affected by egg-laying rate in the current study (*p* > 0.05).

### Antioxidant capacity and apoptosis of ovary and reproductive tract

Ovarian antioxidant capacity showed striking differences between groups (Table 1), the HR group demonstrated significantly elevated levels of antioxidant enzyme activities (CAT, GSH-ST, GSH-Px, and T-SOD) in the ovaries when compared to the LR group (*p* < 0.05). Furthermore, in the uterus, the HR group’s antioxidant enzyme activities (CAT, T-AOC, GSH, GSH-ST, and T-SOD) were also higher than those in the LR group (*p* < 0.05), whereas the lipid peroxidation marker (MDA) showed a decrease in the HR group. Additionally, both the MR and HR groups displayed enhanced activities of GSH, GSH-Px, and T-SOD, with reduced MDA levels in the magnum relative to the LR group (*p* < 0.01).

**Table 1:** The antioxidant capacity of ovary and reproductive tract of laying hens with different egg laying rates.

Item^1^	T-AOC, U/mg	GSH, nmol/mg	MDA, nmol/mg	GSH-ST, U/mg	GSH-Px, U/mg	CAT, U/mg	T-SOD, U/mg
Ovary							
LR	0.62	3.22	3.91^a^	56.29^c^	284.02^b^	170.94^b^	96.59^b^
MR	0.61	5.37	2.57^b^	70.92^b^	330.14^ab^	188.20^b^	119.26^ab^
HR	0.62	4.57	2.97^ab^	86.02^a^	362.44^a^	253.26^a^	120.22^a^
SEM	0.09	0.67	0.36	4.74	17.5	16.87	6.46
*P*-value	0.99	0.10	0.04	<0.01	0.02	<0.01	0.03
Magnum							
LR	0.54	5.02^b^	1.40^a^	105.34	242.16^b^	144.68	76.67^b^
MR	0.64	8.17^a^	0.72^b^	107.73	311.59^a^	142.67	107.82^a^
HR	0.88	7.55^a^	0.66^b^	109.04	273.41^a^	157.15	96.84^a^
SEM	0.14	0.54	0.10	5.89	13.90	7.45	5.99
*P*-value	0.25	<0.01	<0.01	0.90	<0.01	0.35	<0.01
Uterus							
LR	0.38^b^	7.10^b^	3.15^a^	95.29^b^	487.20	111.67^b^	69.50^b^
MR	0.62^ab^	7.43^b^	2.46^b^	118.49^a^	520.54	133.99^a^	74.69^b^
HR	0.72^a^	10.20^a^	1.92^b^	117.14^a^	558.42	130.95^a^	104.22^a^
SEM	0.08	0.74	0.21	4.83	37.36	5.62	8.03
*P*-value	0.02	0.01	<0.01	<0.01	0.37	0.02	0.01

^a,b^Means with different superscripts within a column differ significantly (*P* ≤ 0.05) (*n* = 10).

1 CAT, catalase; T-AOC, total antioxidant capacity; GSH, glutathione; MDA, malondialdehyde; GSH-ST, glutathione S-transferase; GSH-Px, glutathione peroxidase; T-SOD, total superoxide dismutase; LR, low egg laying rate; MR, normal egg laying rate; HR, high egg laying rate.

**Figure 3:**
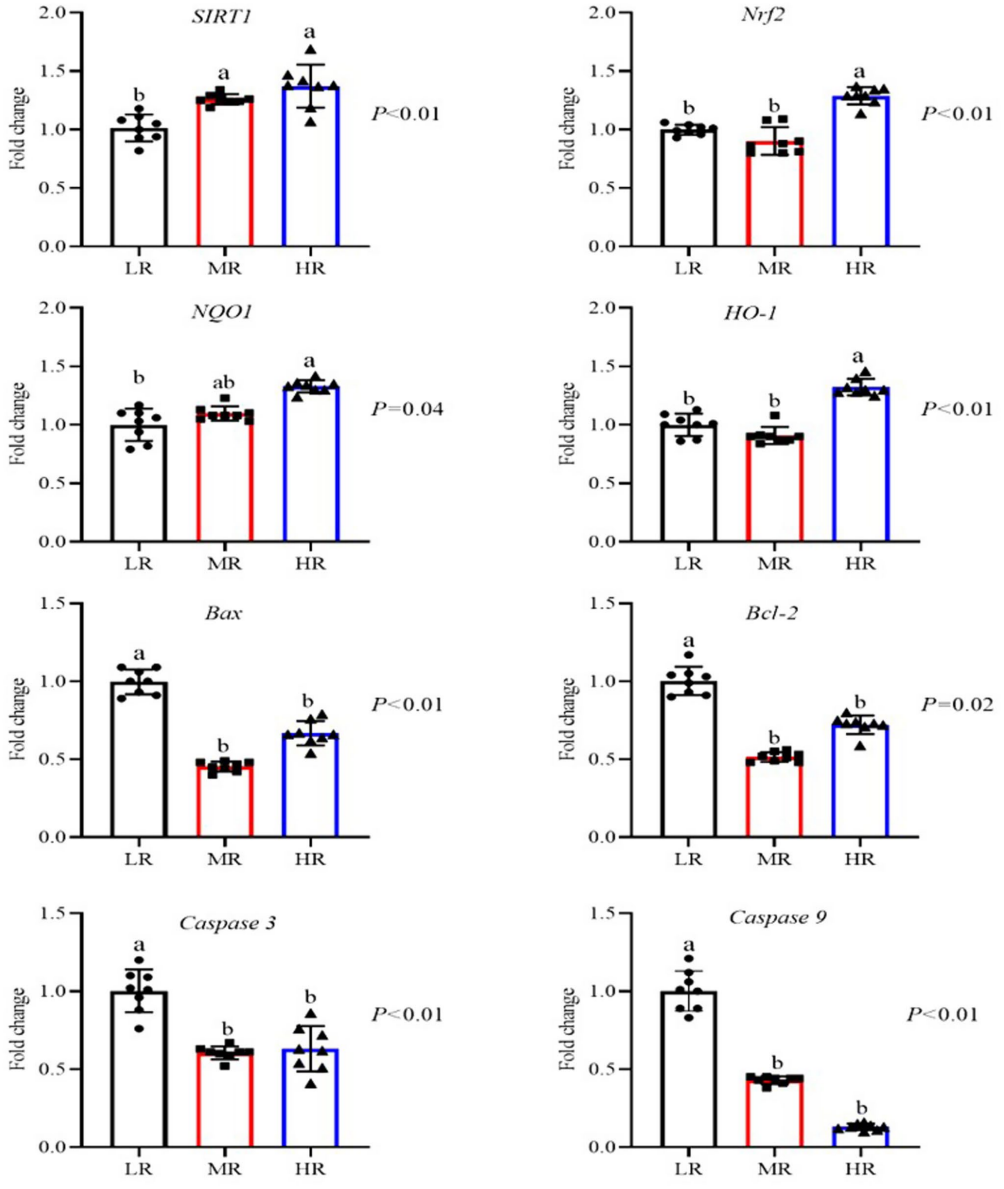
Differential analysis of related genes mRNA expression of ovary in laying hens with different egg-laying rates. LR, low egg laying rate; MR, normal egg laying rate; HR, high egg laying rate; *SIRT1*, sirtuin 1; *Nrf2*, nuclear factor erythroid2-related factor 2; *NQOI*, NAD(P)H: quinone oxidoreductase 1; *HO-1*, heme oxygenase-1; *Bax*, B lymphoma 2 associated X protein; *Bcl-2*, B-cell lymphoma-2; *Caspase 3*, cysteine aspastic 3; *Caspase 9*, cysteine aspastic 9. a, b means with different superscripts within a column differ significantly (*P* ≤ 0.05) (*n* = 8).

**Figure 4:**
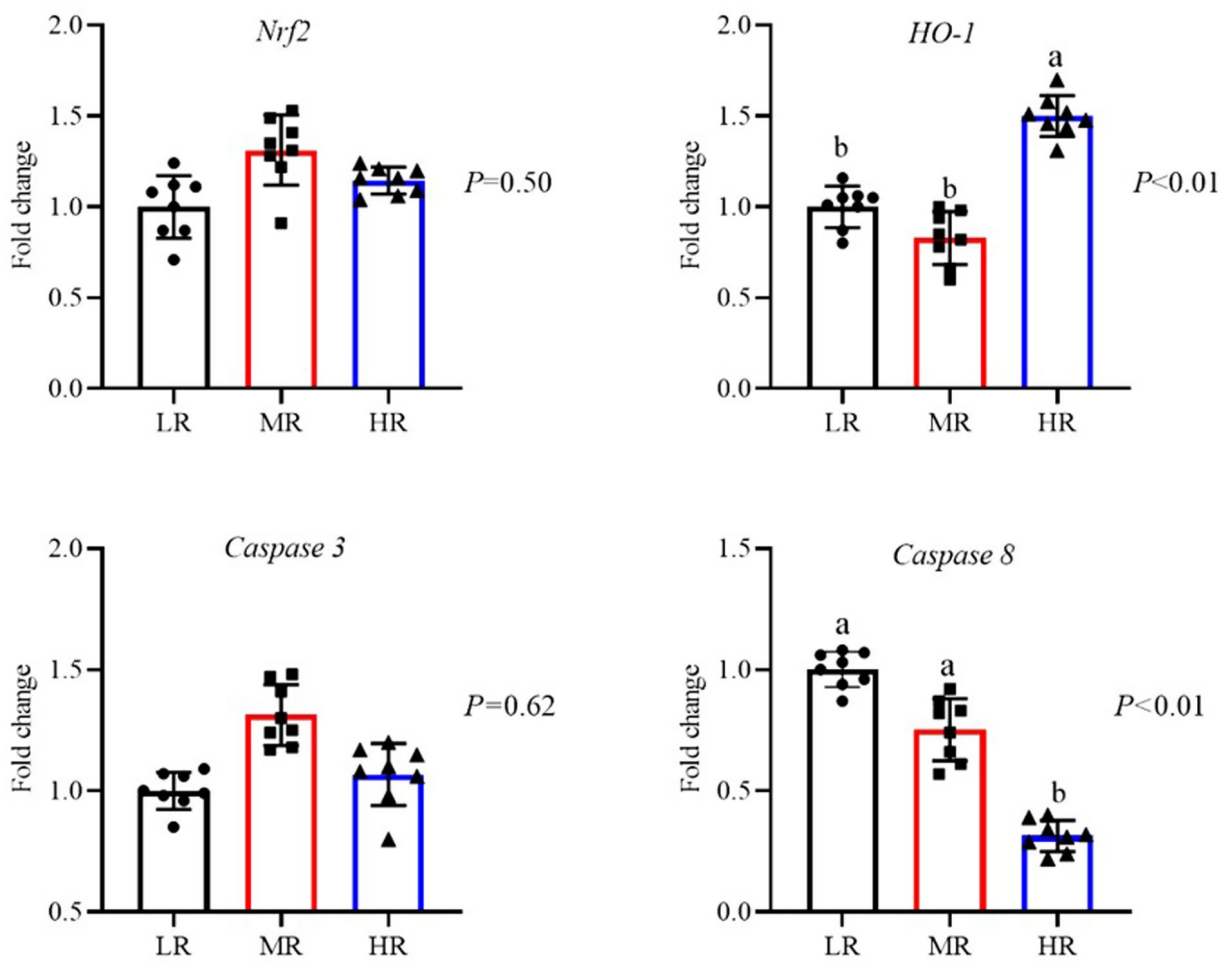
Differential analysis of related genes mRNA expression of magnum of oviduct in laying hens with different egg-laying rates. LR, low egg laying rate; MR, normal egg laying rate; HR, high egg laying rate; *Nrf2*, nuclear factor erythroid2-re1ated factor 2; *HO-1*, heme oxygenase-1; *Caspase 3*, cysteine aspastic 3; Caspase 8, cysteine aspastic 8. a, b means with different superscripts within a column differ significantly (*P* ≤ 0.05) (*n* = 8).

**Figure 5:**
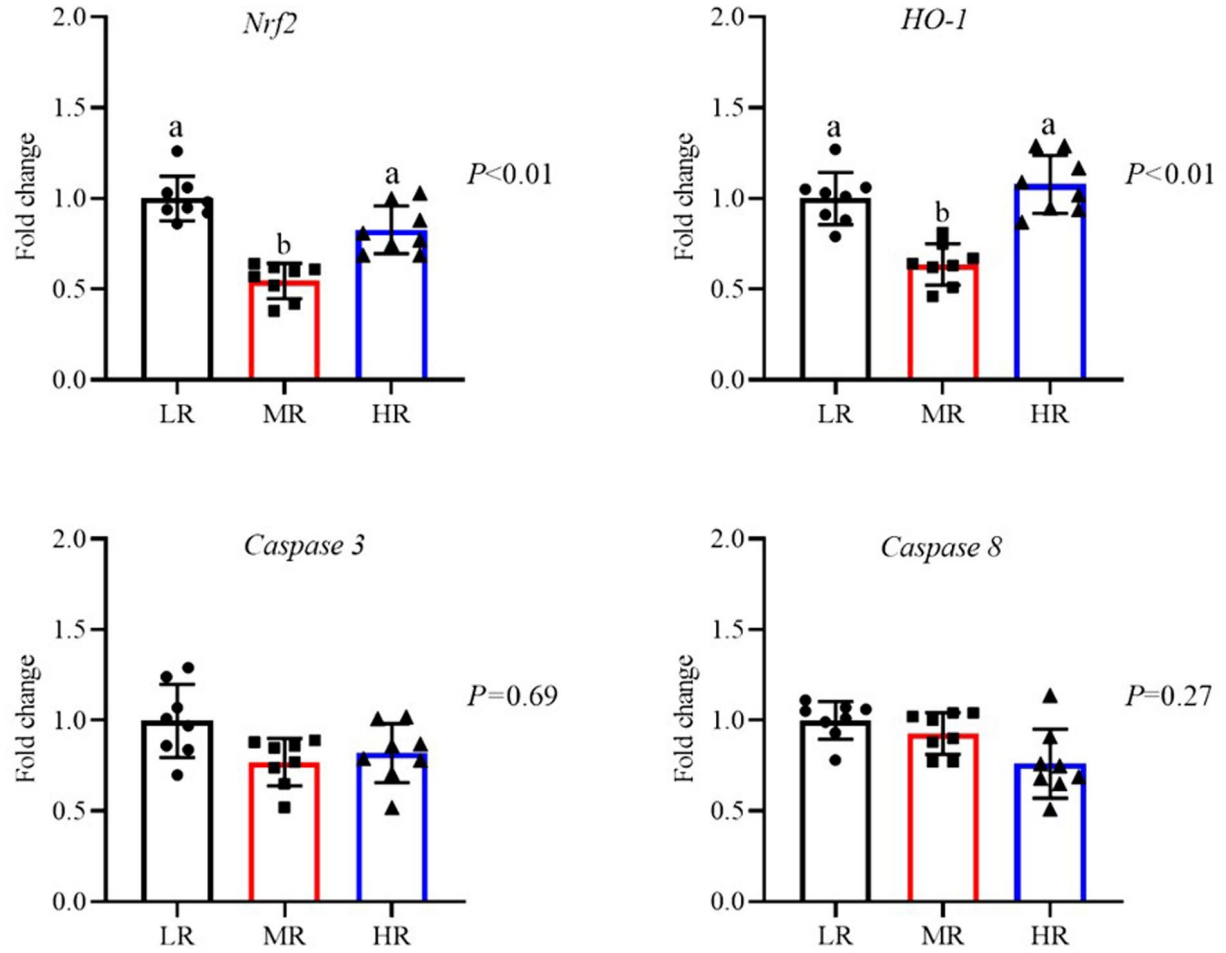
Differential analysis of related genes mRNA expression of uterus in laying hens with different egg-laying rates. LR, low egg laying rate; MR, normal egg laying rate; HR, high egg laying rate; *Nrf2*, nuclear factor erythroid2-related factor 2; *HO-1*, heme oxygenase-1; *Caspase 3*, cysteine aspastic 3; *Caspase 8*, cysteine aspastic 8. a, b means with different superscripts within a column differ significantly (*P* ≤ 0.05) (*n* = 8).

As illustrated in Figure 3, an increased level of *SIRT1* mRNA in the ovaries of layers from both the MR and HR groups (*p* < 0.01). And the expression of mRNA for *Nrf2*, *NQO1*, and *HO-1* was elevated in the HR group when compared to the other groups (*p* < 0.01). In contrast, the MR and HR groups showed reduced mRNA levels of pro-apoptotic factors (*Bax*, *Caspase 3*, and *Caspase 8*) in their ovaries (*p* < 0.05). Furthermore, the anti-apoptotic factor (*Bcl-2*) was decreased in the HR and MR groups. As depicted in Figure 4, compared to the LR group, the HR group’s mRNA levels of *HO-1* were elevated, while *Caspase 8* mRNA levels were decreased in the magnum (*p* < 0.01). Figure 5 illustrates that the *Nrf2* and *HO-1* mRNA expression were higher in the HR and LR groups of the uterus (*p* < 0.01). The mRNA levels of *Nrf2* and *Caspase 3* in the magnum, as well as *Caspase 3* and *Caspase 8* in the uterus, were not statistically affected by varying egg-laying rates (*p* > 0.05).

### Intestinal barrier function

**Figure 6:**
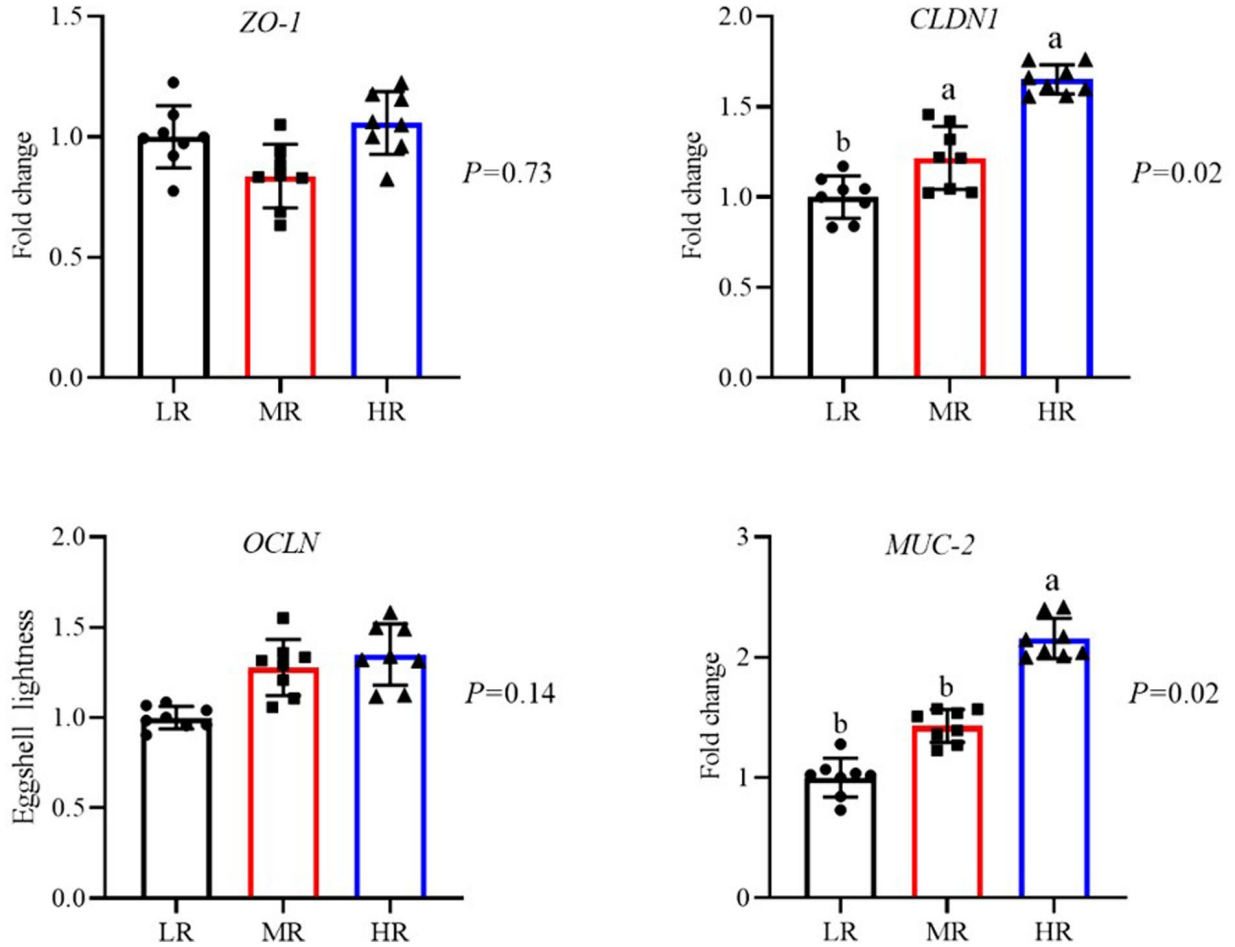
Differential analysis of intestinal barrier function in laying hens with different egg-laying rates. LR, low egg laying rate; MR, normal egg laying rate; HR, high egg laying rate; *ZO-1*, zonula occludens protein-1, *CLDN1*, claudin1; *OCLN*, occludin; *MUC-2*, mucin-2. a, b means with different superscripts within a column differ significantly (*P* ≤ 0.05) (*n* = 8).

Jejunal barrier function differed significantly between groups (Figure 6). The mRNA expression of intestinal barrier-related genes, specifically *CLDN1* and *MUC-2*, was higher in the jejunal mucosa of the HR group compared to the LR group. Additionally, the MR group exhibited up-regulated *CLDN1* mRNA expression relative to the LR group (*p* < 0.05).

### Cecum microbiota composition

**Figure 7:**
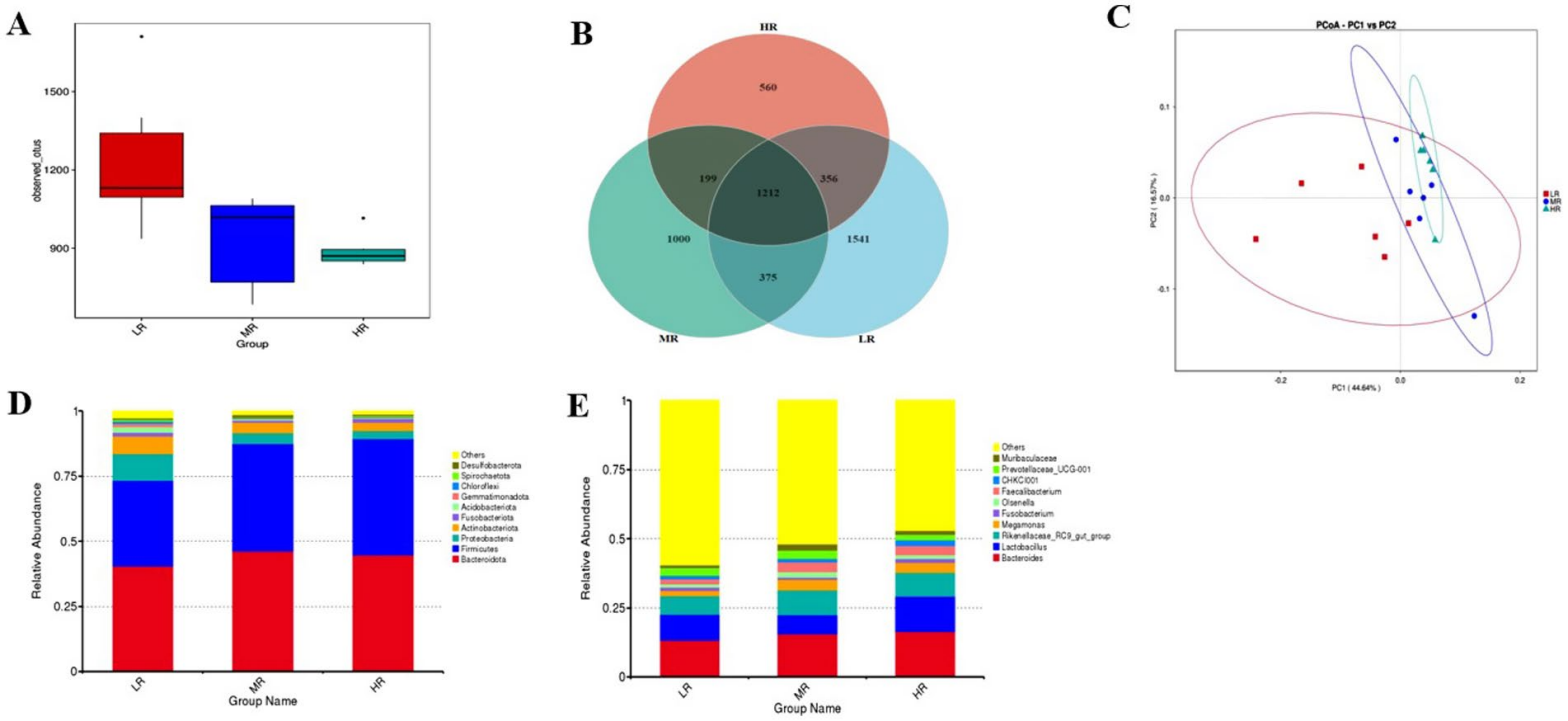
Bacterial OTUs derived from each sample **(A)**. Venn diagram illustrated in cecum microbiota among the samples **(B)**. Principal coordinate analysis plot of the cecum microbiota **(C)**. The relative abundance of the top 10 phylum from samples **(D)**. Bar graph of the top 10 genera from samples **(E)**. Each mean represents I layer/replicate, 6 replicates/treatment. LR, low egg laying rate; MR, normal egg laying rate; HR, high egg laying rate.

**Figure 8:**
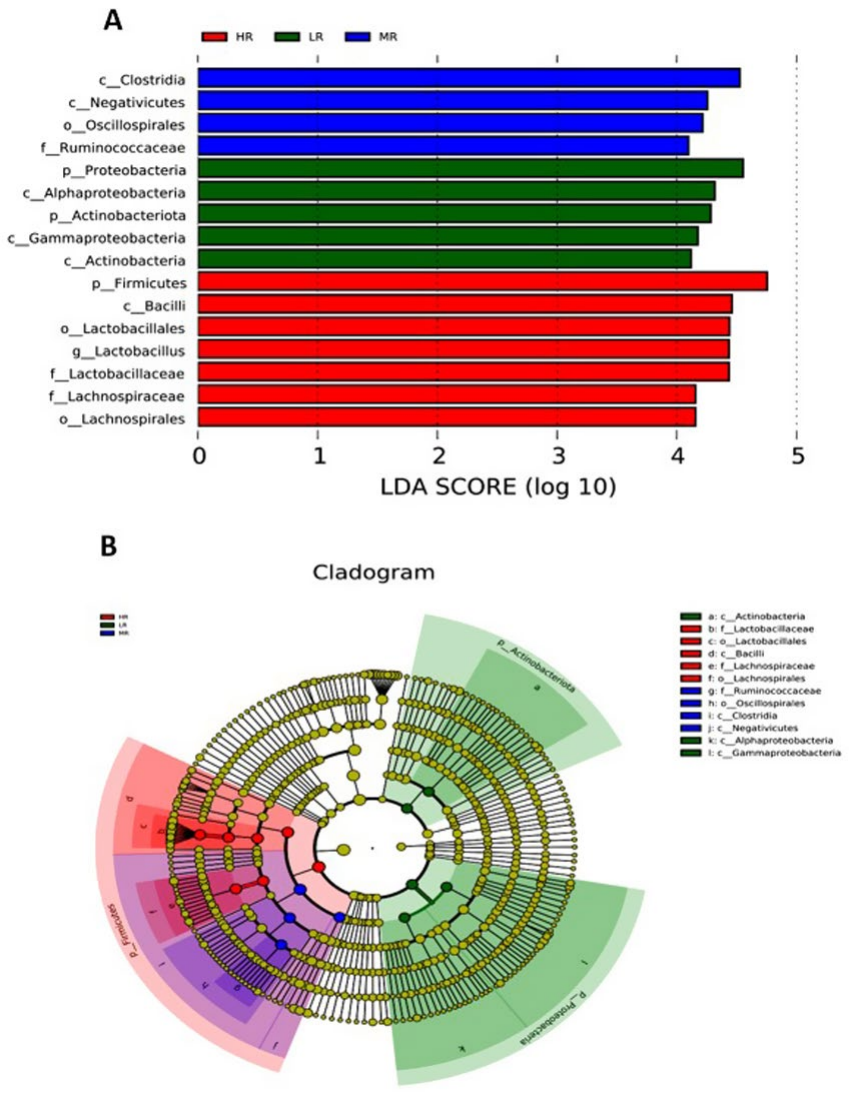
Taxonomic cladogram obtained from LEfSe analysis of 16S rRNA sequencing. Biomarker taxa are heighted by colored circles and shaded areas. Each circle’s diameter is relative to abundance of taxa in the community. Each mean represents I layer/replicate, 6 replicates/treatment. LR, low egg laying rate; MR, normal egg laying rate; HR, high egg laying rate.

Cecal microbiota analysis revealed distinct community structures (Figures 7, 8; Tables 2–4), the abundance of *Firmicutes* (phylum) and *Faecalibacterium* (genus) was increased, while a decrease was observed in the abundance of *Proteobacteria* (phylum) and *Actinobacteriota* (phylum) within the MR and HR groups (*p* < 0.05). Then, the HR group exhibited a significantly higher abundance of *Lactobacillus* (genus) than the MR group (*p* = 0.04). The shared operational taxonomic units (OTUs) among the three groups were depicted in Figure 7B. Furthermore, Figure 7C showed that the distribution of microorganisms in the gut was more concentrated in the HR group. These findings indicated distinct microbial patterns among the three groups.

**Table 2:** Relative abundances of top 5 microbiota at phylum level of laying hens with different egg laying rates.

Item, %^1^	Bacteroidota	Firmicutes	Proteobacteria	Actinobacteriota	Fusobacteriota
LR	40.50	32.83^b^	10.17^a^	7.00^a^	1.33
MR	46.17	41.17^a^	4.17^b^	4.00^b^	0.67
HR	45.00	44.33^a^	3.17^b^	3.17^b^	1.33
SEM	0.03	0.02	0.01	<0.01	<0.01
*P*-value	0.32	<0.01	<0.01	<0.01	0.76

^a,b^Means with different superscripts within a column differ significantly (*P* ≤ 0.05) (*n* = 10).

1 LR, low egg laying rate; MR, normal egg laying rate; HR, high egg laying rate.

**Table 3:** Relative abundances of top 5 microbiota at genus level of laying hens with different egg laying rates.

Item, %^1^	Bacteroides	Lactobacillus	Rikenellaceae	Megamonas	Faecalibacterium
LR	13.17	9.33^ab^	6.83	1.83	2.00^b^
MR	15.83	6.83^b^	9.00	3.67	3.50^a^
HR	16.50	12.67^a^	8.67	3.33	3.50^a^
SEM	0.02	0.01	0.01	<0.01	<0.01
*P*-value	0.31	0.04	0.29	0.37	0.02

^a,b^Means with different superscripts within a column differ significantly (*P* ≤ 0.05) (*n* = 10).

1 LR, low egg laying rate; MR, normal egg laying rate; HR, high egg laying rate.

**Table 4:** Differential analysis of alpha diversity index in laying hens with different egg laying rates.

Item^1^	Observed_otus	Shannon	Simpson	Chao1
Groups				
LR	1254.00^a^	8.94^a^	1.00^a^	1256.00^a^
MR	940.00^b^	8.36^b^	0.99^b^	942.00^b^
HR	900.00^b^	8.28^b^	0.99^b^	903.20^b^
SEM	83.50	0.13	<0.01	83.83
*P*-value	0.02	<0.01	0.02	0.02

^a,b^Means with different superscripts within a column differ significantly (*P* ≤ 0.05) (*n* = 8).

1 LR, low egg laying rate; MR, normal egg laying rate; HR, high egg laying rate.

### Alpha and beta diversity of cecum microbiota

Microbial diversity analysis revealed distinct patterns among the experimental groups (Table 4). The MR and HR groups exhibited significantly lower observed OTUs, Shannon, Simpson, and Chao1 indices in the cecal microbiota (*p* < 0.05). Figure 8 illustrated that the microbial species composition was significantly different in the cecal chyme of layers, with a more concentrated microbial distribution observed in the HR group of laying hens. LEfSe analysis indicated that the MR group had a higher relative abundance of *Clostridia* (class), *Negativicutes* (class), and *Oscillatoriales* (order), while the HR group showed a higher relative abundance of *Firmicutes* (phylum), *Bacilli* (class) and *Lactobacillales* (order) in the cecum (LDA score > 4, *p* < 0.05).

### Correlations between cecum microbiota and production performance of layers

**Figure 9:**
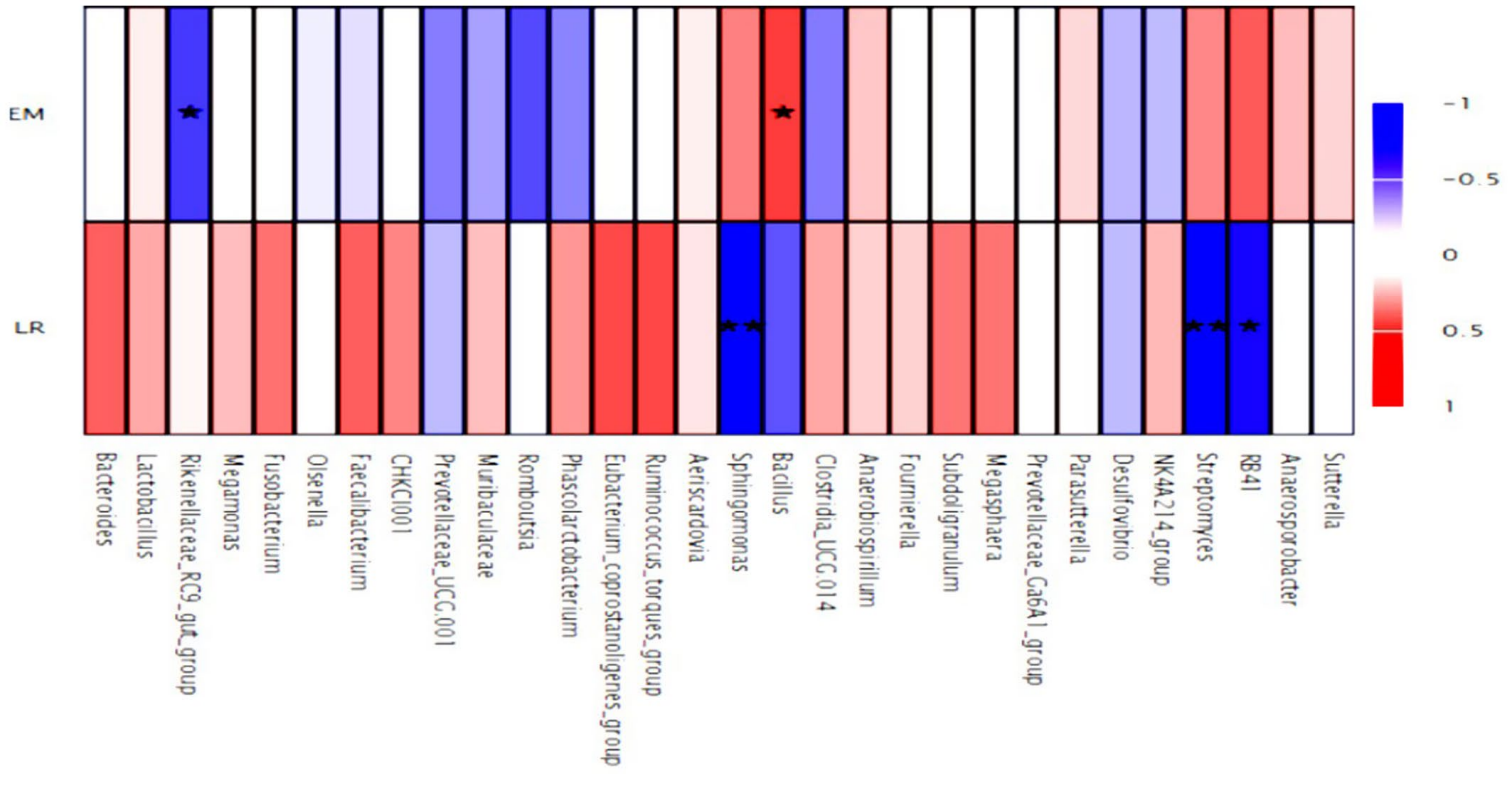
Heatmap of the spearman correlations between the gut microbiota significantly modified by different egg laying rates at genus level (top 30). Red indicates positive correlation, and blue indicates negative correlation; while the color is darker, the correlation is higher. *P < 0.05 and **P < 0.01 (following Spearman correlation analysis, the correlation *p*-values underwent false discovery rate (FDR) correction using the Benjamini-Hochberg procedure). Each mean represents 4 layers/replicate, 10 replicates/treatment. LR, egg laying rate; EM, egg mass.

This study explored the correlations between gut microbiota at the genus level and various parameters of production performance, including egg-laying rate and egg mass in Figure 9. The results of the correlation analysis indicated that the genera *RB41*, *Streptomyces*, and *Sphingomonas* were negatively correlated with the egg-laying rate. In contrast, *Bacillus* demonstrated a positive correlation with egg mass, while *Rikenellaceae_RC9_gut_group* was negatively correlated with egg mass in laying hens.

## Discussion

Various elements affect the reproductive capabilities of hens, such as genetic background, age, nutrition, and environmental factors (Bova et al., 2014; Rozenboim et al., 2007; Shi et al., 2020). In our research, we noted that the HR group demonstrated greater egg production, egg mass, and quality egg rates, alongside a reduced FCR, even though both sets of hens were under identical conditions (genetic background, age, nutrition, management, and environment). Previous studies have similarly indicated that breeders with higher egg-laying rates tend to have higher qualified egg rates and lower FCR (Wang J. et al., 2021; Zhao et al., 2019). But this experiment was conducted exclusively on a single breed of post-peak laying hens with a limited sample size. Future studies should expand the population diversity (different breeds and ages) to validate the generalizability of the findings. It is well established that the ovary serves as a critical reproductive organ in females, where the atresia and apoptosis of follicles occur (Yu et al., 2004). Pro-apoptotic factors primarily mediate the transmission of cell death signals (Li and Yuan, 2008; Melo et al., 2015). In our study, the HR group demonstrated lower mRNA expression levels of *Bcl2*, *caspase 3*, and *caspase 8* in both the ovary and oviduct. A related study found that breeders with superior reproductive performance exhibited reduced mRNA expression of *caspase 9* and *Bax* (Wang J. et al., 2021). Additionally, research found that the mRNA levels of *caspase 9* was increased in early and advanced atretic follicles (Matsui et al., 2003). These findings suggested that laying hens with higher egg-laying rates possessed less atretic follicles in the ovary and oviduct, enabling enhanced production performance during later laying stages.

The egg quality is closely associated with age in hens (Gu et al., 2021). There is a significant decline in eggshell quality of older hens (Bain et al., 2016). Additionally, albumen quality, a crucial aspect of internal egg quality, also decreases over time (Brenes et al., 2008; Reis et al., 2019). In our research, the HR group showed greater eggshell strength and Haugh units. This result aligns with our earlier studies, which indicated that broiler breeders or laying hens with elevated egg-laying rates typically possess enhanced egg quality, characterized by thicker eggshells and a higher eggshell ratio (Qiang et al., 2023; Zhao et al., 2019). Nonetheless, there are few studies examining the differences in albumen quality among laying hens with varying laying levels. Given that eggshell and albumen quality are formed in the uterus and magnum of the oviduct in laying hens (Wang J. et al., 2021), which is essential for egg quality. Oxidative stress is a common occurrence in animals (Ding et al., 2022). In response to this stress, the body upregulates the expression of antioxidant enzymes and related genes to enhance its antioxidant capacity. In this investigation, we found that the HR group showed greater activities of antioxidant enzymes (CAT, T-AOC, GSH, GSH-ST, and T-SOD) and enhanced expression of related genes (*SIRT1*, *Nrf2*, *NQO1*, and *HO-1*) in both the ovary and oviduct. Supporting our results, Wang J. et al. (2021) noted that breeders with higher egg-laying rates exhibited elevated activities of antioxidant enzymes (SOD and T-AOC) in the ovary. This relationship may be reinforced by earlier research by Tang et al. (2014), which indicated that *SIRT1* can alter the structure of *Keap1*, thereby activating *Nrf2* and promoting the expression of *NQO1*, *HO-1*, and GSH-ST. Additionally, Huang et al. (2015) found that *SIRT1* could directly activate *Nrf2* expression by facilitating the deacetylation of *Nrf2*, potentially leading to an increased antioxidant capacity. Consequently, these results suggested that laying hens with higher egg-laying levels had superior antioxidant capacity, likely attributed to elevated *SIRT1* levels, which might contribute to their enhanced eggshell and albumen quality. While we identified associations between antioxidant capacity (*SIRT1* and *Nrf2* pathways) and egg quality improvements, the causal mechanisms remain underexplored due to the absence of functional validation through gene knockout or overexpression models or targeted antioxidant interventions.

Improved gut function is broadly recognized as essential for boosting the production performance in hens, given that the gut significantly contributes to nutrient digestion and absorption (Cao et al., 2023; Liu et al., 2016; Wu et al., 2018). The expression levels of specific proteins are generally regarded as important indicators of intestinal function (Cao et al., 2023). Our research found that the HR group exhibited higher *CLDN1* and *MUC-2* mRNA expression in the jejunum. Additionally, our previous studies indicated that the content of the related protein ZO-2 was lower in hens with diminished production performance (Cao et al., 2023). Thus, we speculated that the HR group demonstrated superior intestinal function, with more efficient digestion and absorption, and enhanced production performance. Consistent with our previous research, broiler breeders with higher egg production levels exhibited a higher gut utilization of energy in the feed (Yang et al., 2020). The variety of factors that influence the distribution of intestinal microbiota includes genetics, environmental conditions, diseases, medications, and diet (Bain et al., 2016; Chen et al., 2021). Our findings revealed a more concentrated distribution of microbes in the cecal chyme of the HR group. These results suggested that hens with higher egg-laying performance possessed improved intestinal barrier function and a more favorable distribution of intestinal microbiota, thereby enhancing the digestion and absorption of feed.

The integrity structure of the intestinal microbiota serves as the primary defense barrier, preventing the invasion of pathogenic bacteria (Cheng et al., 2020; Sau et al., 2021; Wang et al., 2020). The predominant microbial species of *Bacteroides*, *Firmicutes*, and *Proteobacteria* were found in the intestinal tract. The LEfSe analysis indicated that the HR group exhibited higher relative abundances of *Firmicutes*, whereas the relative abundances of *Actinobacteria* and *Proteobacteria* were found to be lower. Previous studies have also shown that high-laying rate breeders possess a greater abundance of *Firmicutes* and *Bacteroidetes*, whereas obese animals and humans tend to exhibit lower *Firmicutes* abundances and higher *Bacteroidetes* abundances (Levy et al., 2016; Ma et al., 2018; Cao et al., 2023). *Bacteroidetes* and *Lactobacillus* are typically seen as advantageous bacteria. Therefore, the findings from this experiment revealed that the HR group possessed a greater proportion of beneficial bacteria and displayed a more favorable composition of gut microbiota. Consequently, the HR group showed a more intact structure of microbiota, leading to better nutrient utilization and improved performance in production. The microbial profile of the HR group (*Firmicutes*, *Lactobacillus* and *Faecalibacterium*) indicated enhanced metabolic efficiency and intestinal barrier function. Butyrate derived from *Faecalibacterium* may suppress oviductal inflammation through inhibition of the NF-κB pathway, which is consistent with the observed ovarian *caspase 3* and uterine MDA reductions. Concurrently, Lactobacillus-mediated upregulation of mucin-2 likely fortifies intestinal barrier integrity, thereby mitigating endotoxin translocation that exacerbates systemic oxidative stress.

The gut microbiota is important for the development and growth of laying hens (Lunedo et al., 2019; Wang J. M. et al., 2021). We observed a negative correlation between harmful bacteria, specifically the genera *Streptomyces* and *Sphingomonas*, and the egg-laying rate of these hens in study. Conversely, *Bacillus* demonstrated a positive correlation with egg mass. *Bacillus* is classified as beneficial. Therefore, the makeup and variety of gut microbiota can greatly affect the production efficiency in older hens. Nevertheless, fecal microbiota transplantation (FMT) can also be used to verify the correlation between gut microbiota profile (Firmicutes) and production performance to establish a causal relationship in the future.

## Conclusion

Overall, our findings suggested that layers with a higher egg-laying rate exhibited enhanced productive performance and egg quality, which may be associated with superior intestinal health (better barrier function and microbiome structure) and better function of the reproductive tract (improved antioxidant capacity). Additionally, the *SIRT1*-related apoptosis and *Nrf2* signaling pathways may contribute to improved ovarian function. The superior functions of intestinal, reproductive tract and ovaries facilitated better digestion and absorption of nutrients, as well as improvements in production performance (see Graphical abstract).

## Data Availability

The data presented in the study are deposited in the NCBI Trace Archive NCBI Sequence Read Archive repository, accession number PRJNA1279077.
