# Supplementary material for: Comparative analysis of intestinal and reproductive function in older laying hens with three egg laying levels
Source: Front Microbiol. 2025 Jul 3;16:1582516. doi: 10.3389/fmicb.2025.1582516 (PMC12269605; doi:10.3389/fmicb.2025.1582516)
Supplement: Supplementary file 1 [file Table_1.docx]

| **Supplementary Table 1**  Composition and nutrient level of basal diet (as-fed basis). | |
| --- | --- |
| Item | Amount, % |
| Corn | 62.06 |
| Soybean meal | 25.55 |
| Soybean oil | 1.00 |
| Calcium carbonate | 9.50 |
| Calcium hydrophosphate | 1.00 |
| L-Lysine HCl | 0.05 |
| DL-Methionine | 0.20 |
| Threonine | 0.02 |
| NaCl | 0.25 |
| Sodium bicarbonate | 0.10 |
| Choline chloride | 0.10 |
| Vitamin and mineral premix^1^ | 0.17 |
| Total | 100.00 |
| Analyzed nutrient levels, % |  |
| ME^2^, kcal/kg | 2725.00 |
| Crude protein | 16.11 |
| Calcium | 3.98 |
| Total phosphorus | 0.51 |
| Lysine | 0.80 |
| Methionine | 0.42 |
| ^1^Provided per kilogram of diet: vitamin A, 9300 IU; vitamin D3, 3,000 IU; vitamin E, 30 IU; vitamin K3, 4.8 mg; thiamin, 3.0 mg; riboflavin, 9.6 mg; pyridoxine, 6.0 mg; vitamin B12, 0.3 mg; folic acid, 1.5 mg; niacin, 60 mg; pantothenic acid, 18 mg; biotin, 1.67 mg; iron, 60 mg; copper, 8 mg; manganese, 60 mg; zinc, 80 mg; selenium, 0.30 mg; iodine, 0.35 mg.  ^2^Calculated according to NRC (1994). | |

| **Supplementary Table 2**  Related gene and primer information^1^. | | | | |
| --- | --- | --- | --- | --- |
| Genes | Primer sequences (5'-3') | | Product size | Accession no. |
| *Β-actin* | Forward | GCTACAGCTTCACCACCACA | 90 | NM-205518.1 |
|  | Reverse | TCTCCTGCTCGAAATCCAGT |  |  |
| *Bax* | Forward | GTACGTCAATGTGGTCACCC | 210 | XM-015274882 |
|  | Reverse | TGGGATAATGCTGGGGTTGA |  |  |
| *Bcl2* | Forward | GCCTTTGTGGAATTGTACGG | 124 | [NM-205339.3](https://www.ncbi.nlm.nih.gov/nuccore/NM_205339.3) |
|  | Reverse | GTCCAAGATAAGCGCCAAGA |  |  |
| *Caspase 3* | Forward | AAAGATGGACCACGCTCAGG | 204 | NM-204725 |
|  | Reverse | TGAACGAGATGACAGTCCGG |  |  |
| *Caspase 8* | Forward | CCGGACGTTTTCATTGAGAT | 108 | NC-052538.1 |
|  | Reverse | CTCTTGTCCACTTTGCCACA |  |  |
| *Caspase 9* | Forward | TATGGTGGAGGACATGCAGA | 99 | XM-424580.5 |
|  | Reverse | AATATTGGGAAGGCCTGCTT |  |  |
| *CLDN1* | Forward | TGGGTCTGGTTGGTGTGTTT | 108 | NM-001013611.2 |
|  | Reverse | AGATCACGCCACCAAAGACA |  |  |
| *HO-1* | Forward | CATGCCTACACCCGCTATTT | 104 | X56201.1 |
|  | Reverse | AAAGCCAACCCTTCTCCAGT |  |  |
| *MUC-2* | Forward | ACCAAGCAGAAAAGCTGGAA | 80 | NM-001318434.1 |
|  | Reverse | AAATGGGCCCTCTGAGTTTT |  |  |
| *NQO1* | Forward | AACCCCGAGTGCTTTGTCTA | 101 | NM-001277620.2 |
|  | Reverse | GCCGCTTCAATCTTCTTCTG |  |  |
| *Nrf2* | Forward | ACGGTGACACAGGAACAACA | 87 | MN416129.1 |
|  | Reverse | ACAGCGGGAAATCAGAAAGA |  |  |
| *OCLN* | Forward | GCTGAGATGGACAGCATCAA | 97 | NM-205128.1 |
|  | Reverse | TGCCACATCCTGGTATTGAG |  |  |
| *SIRT1* | Forward | TAGCCAATGGTTTCCACTCC | 149 | NM-001004767.2 |
|  | Reverse | AAGAATTGTCCGTGGGTCTG |  |  |
| *ZO-1* | Forward | GGCAAGTTGAAGATGGTGGT | 135 | XM-015278981.2 |
|  | Reverse | ATGCCAGCGACTGAATTTCT |  |  |
| ^1^Abbreviations: *CLDN1* = claudin 1; *Cyt-C* = cytochrome c; *HO-1* = heme oxygenase-1; *MUC2* = mucin-2; *NQO1* = NAD(P)H: quinone oxidoreductase 1; *Nrf2* = nuclear factor erythroid 2-related factor 2; *OCLN* = occludin; *TNF-α* = tumor necrosis factor alpha; *ZO-1* = zonula occludens protein-1. | | | | |
