# Supplementary figures and images for: Comparative analysis of intestinal and reproductive function in older laying hens with three egg laying levels
Source: Front Microbiol. 2025 Jul 3;16:1582516. doi: 10.3389/fmicb.2025.1582516 (PMC12269605; doi:10.3389/fmicb.2025.1582516)

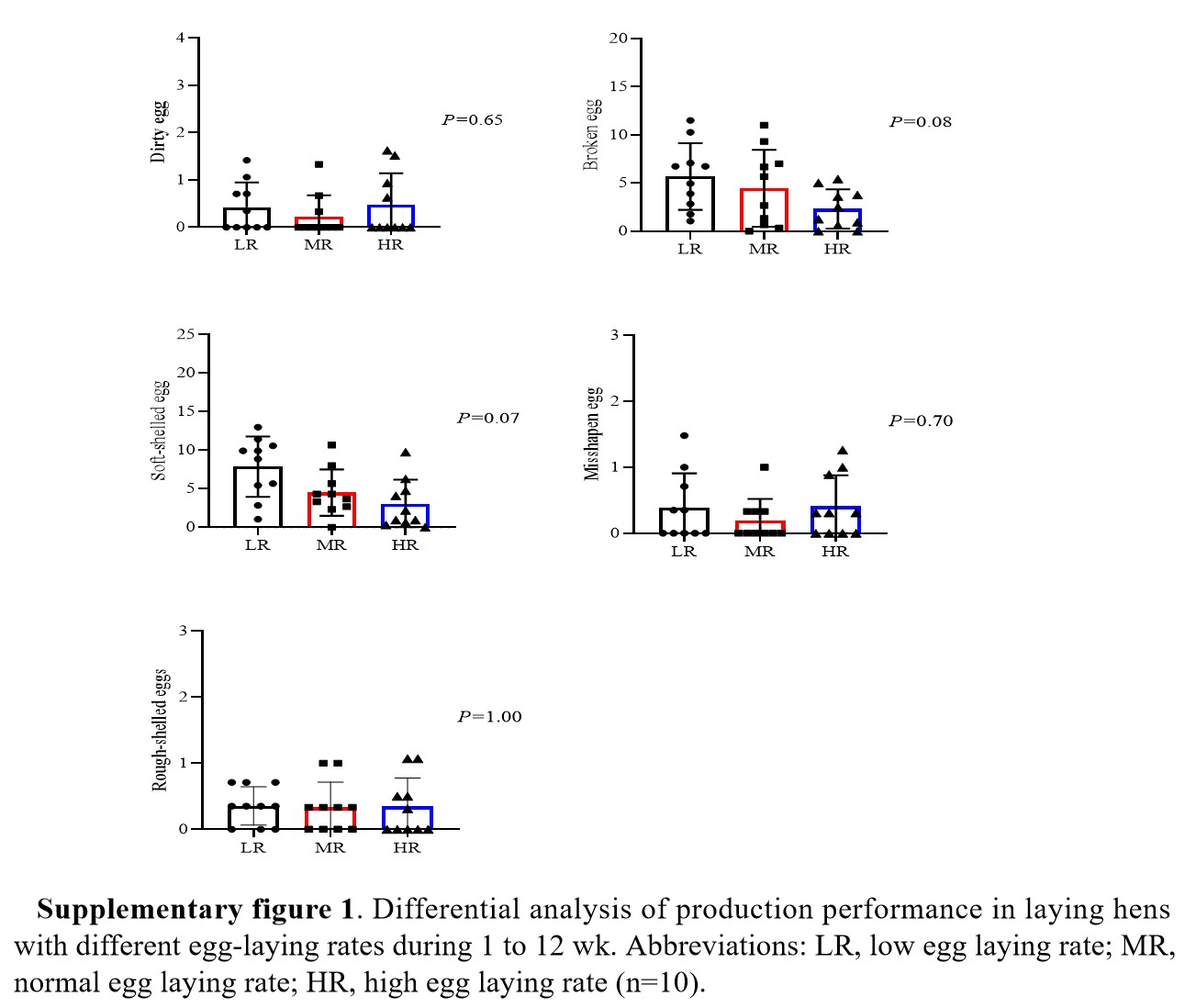

Supplement: Supplementary file 2 [file Image_1.jpg]
